# Arabinoxylan Improves Quality and Inhibits Starch Retrogradation in Mashed Potatoes Under Cold Storage

**DOI:** 10.3390/foods15122212

**Published:** 2026-06-19

**Authors:** Siyu He, Xinyi Luo, Zifan Zhao, Liang Li, Jiahong Duan, Shang Lin, Wen Qin

**Affiliations:** College of Food Science, Sichuan Agricultural University, Ya’an 625014, China; hesiyu479@stu.sicau.edu.cn (S.H.); 2024418095@stu.sicau.edu.cn (X.L.); 2024218014@stu.sicau.edu.cn (Z.Z.); 202205281@stu.sicau.edu.cn (L.L.); 202205278@stu.sicau.edu.cn (J.D.)

**Keywords:** arabinoxylan, mashed potatoes, starch retrogradation, storage quality

## Abstract

Mashed potatoes (MP) are widely consumed starch-based foods. However, their shelf life is limited by starch retrogradation during low-temperature storage, which causes texture hardening, water exudation, and sensory deterioration. Although natural polysaccharides can modulate starch properties, the specific anti-retrogradation effect of soluble arabinoxylan (AX) in complex MP matrices remains unknown. In this study, the effects of AX on the physicochemical and sensory qualities of MP during 7 d of storage at 4 °C were comprehensively investigated. Results demonstrated that AX significantly reduced the rheological moduli (i.e., G′ and G″ values) and hardness of stored MP. Additionally, LF-NMR, XRD, FTIR and SEM analyses, together with water holding capacity (WHC) measurement, revealed that AX improved water retention and restricted water mobility of the system, delayed starch recrystallization, inhibited the formation of short-range ordered structures, and physically disrupted the starch microstructure, thereby attenuating the overall starch retrogradation process. Moreover, the addition of AX helped maintain the sensory appeal of the products. These findings suggest that AX modulates the structural evolution of the starch matrix during storage. This distinguishes the present work from conventional hydrocolloid studies by demonstrating that AX can simultaneously inhibit starch retrogradation, stabilize color, and maintain soft texture. This work highlights the potential of AX as a clean-label multifunctional modifier to extend the shelf life of starchy convenience foods.

## 1. Introduction

Mashed potatoes (MP) are convenient, semi-solid starch-based foods that are widely favored by consumers due to their soft texture and easy consumption [1]. One of the most common MP forms is dehydrated powder, which requires manual reconstitution. The powdered MP products cannot satisfy the increasing consumer demand for immediate convenience and yield inconsistent hydration. Hence, the ready-to-eat MP products on the market aim to directly provide an ideal soft, smooth, and homogeneous texture without additional preparation processes.

Despite their convenience, preserving the quality of ready-to-eat MP remains challenging. Owing to the growing consumer preference for clean-label foods, i.e., minimal addition of chemical preservatives, cold-chain storage (typically at 4 °C) has become the primary preservation strategy. However, existing studies have confirmed that low-temperature storage can induce severe quality deterioration, which is predominantly caused by starch retrogradation [2,3]. Since starch accounts for 60–80% of the dry matter in MP, its molecular rearrangement and recrystallization during cold storage rapidly degrade the food matrix. The starch retrogradation of MP products may impair the desired creamy texture and cause continuous hardening of the gel network, subsequent syneresis, and overall sensory decline [4,5]. Consequently, shelf-life extension and industrial application of ready-to-eat MP products are limited.

To alleviate the starch retrogradation issue without compromising food safety, natural polysaccharides have been widely applied to optimize gel properties and inhibit starch retrogradation in starch-based food systems [6]. Previous studies suggest that polysaccharides may interact with starch molecules mainly via hydrogen bonding and steric hindrance. Such interactions can restrict the movement and spatial rearrangement of starch chains as well as their ordered crystallization, thus maintaining a favorable gel texture and retarding starch retrogradation [7,8].

Among various plant-derived hydrocolloids, arabinoxylan (AX)—the most abundant hemicellulose in cereal bran and endosperm—has garnered significant attention, accounting for up to 70% of the total dietary fiber in these tissues [9]. AX possesses a backbone composed of *β*-(1,4)-D-xylopyranosyl residues, and its side chains are commonly bound to α-L-arabinofuranosyl residues through α-1,2- and/or α-1,3-glycosidic bonds to form mono- or di-substituted branched structures [10]. Structural characteristics of AX, including molecular weight, branching degree, substitution mode and ferulic acid content, vary greatly with cereal origins and extraction techniques [11]. Moreover, such structural diversity offers a potential molecular basis for its interactions with starch constituents and further modulation of related functional characteristics.

Recent studies have demonstrated the regulatory effects of AX on starch gel properties and starch retrogradation mitigation. For instance, Hou et al. reported that AX could inhibit amylose leaching, disrupt intermolecular hydrogen bond networks and hinder amylopectin recrystallization, thus lowering the storage (G′) and loss (G″) moduli of starch gels [12]. Similarly, Dong et al. confirmed that the addition of 0.5% AX with appropriate molecular weight and branching degree significantly reduced the hardness of barley starch gels by blocking the formation of starch double helices and ordered crystal structures [13]. Furthermore, existing research has shown that AX is capable of modulating starch gel properties and retrogradation behavior, providing novel strategies for quality optimization of starch-based products [14,15,16].

Despite the reported regulatory effects of AX in pure starch systems, it is worth noting that most existing studies on AX and other common hydrocolloids (i.e., xanthan gum, guar gum, pectin) have notable limitations. First, most relevant studies were carried out in pure starch matrices, ignoring the influences of endogenous components, including proteins and lipids, in actual complex food systems such as MP. These components may change the interaction modes between starch and polysaccharides. Furthermore, conventional hydrocolloids usually mitigate starch retrogradation by strengthening the gel network, which tends to compromise the inherent soft texture of starch-based foods.

Meanwhile, current research on MP mainly focuses on potato variety screening, nutritional fortification and flavor improvement, while systematic studies concerning texture modification and storage deterioration during cold storage are relatively scarce [17,18]. Therefore, it remains to be systematically investigated whether the steric hindrance and structural modulation effects observed in model starch systems can achieve texture regulation and shelf-life extension in actual MP matrices, without impairing the favorable soft mouthfeel of finished products.

To fill these research gaps, the present study introduced AX, a cereal-derived clean-label dietary fiber, into ready-to-eat MP made from potato flakes. Unlike conventional hydrocolloids, AX mitigates retrogradation without hardening the texture, while its feruloyl groups contribute to color stability.

This study systematically investigated the effects of AX at different addition levels on the macroscopic qualities (i.e., rheological characteristics, texture, water holding capacity, color and sensory attributes) and microstructural variations (i.e., water distribution, crystal structure, ordered structure and micromorphology) of MP during cold storage at 4 °C. In addition, the quality of AX-supplemented MP was compared with commercial ready-to-eat MP products to verify its practical application potential. By clarifying the regulatory mechanism of AX within complex MP matrices, this work intends to offer a novel clean-label approach for developing high-quality cold-chain convenience starch-based foods.

## 2. Materials and Methods

### 2.1. Materials

Potato flakes with starch purity of 76.32 ± 3.52% and amylose content of 15.86 ± 0.67% were supplied by Zhangjiakou Hongji Agricultural Technology Development Co., Ltd., Zhangjiakou, China. Three kinds of commercial instant MP products for quality comparison were separately purchased from Chongqing Meilexian Trading Co., Ltd. (Chongqing, China), Chongqing Haoxifu Food Co., Ltd. (Chongqing, China) and Rizhao Aiwennong Food Co., Ltd. (Rizhao, China). Based on the ingredient lists marked on product packages, commercial MP sample 1 and sample 2 were mainly composed of potatoes, drinking water, vegetable oil and edible salt, while sample 3 was prepared solely from potatoes. None of the three commercial products contained any labeled stabilizers, thickeners or hydrocolloids. These samples were chosen to cover mainstream market categories, including pure potato products and lightly seasoned products, which could well represent prevailing commercial instant MP products. Nevertheless, detailed raw material ratios and specific processing techniques of these commercial products were not publicly disclosed by their manufacturers.

The AX adopted in this study was extracted from corn bran via xylanase hydrolysis. The xylanase was derived from *Aspergillus oryzae* with an enzyme activity of 2.5 U/mg (EC 3.2.1.8) and purchased from Sigma-Aldrich, St. Louis, MO, USA. Briefly, corn bran was pretreated with 95% ethanol to remove phenolic compounds and soluble sugars, and residual starch was further removed by high-temperature α-amylase (Shanghai Macklin Biochemical Co., Ltd., Shanghai, China). After washing and drying, the pretreated material was suspended in 50 mM sodium acetate buffer (pH 6.0) and enzymatically hydrolyzed with xylanase at 30 °C for 24 h. The supernatant was harvested by centrifugation and concentrated, followed by trypsin (Shanghai Macklin Biochemical Co., Ltd., Shanghai, China)-assisted deproteinization. Subsequently, the solution was heated at 100 °C for 15 min to fully inactivate residual enzymes. After cooling and recentrifugation, the supernatant was purified through ethanol precipitation, dialysis (3.5 kDa molecular weight cutoff) and freeze-drying to acquire purified AX. All preparation and structural characterization procedures were conducted according to our previously reported methods [19].

The main structural characteristics of AX are as follows: the extraction yield was 1.54 ± 0.04%, total sugar content was 84.19 ± 0.91%, average molecular weight was 117.40 kDa, and water solubility reached 91.33 ± 0.67%. The apparent viscosity of 1% (*w*/*v*) AX aqueous solution was 199.4 mPa·s at the shear rate of 0.01 s^−1^. Arabinose and xylose were the predominant monosaccharide constituents, which collectively occupied 78.09 ± 0.40 mol% of total monosaccharides, and the corresponding branching degree was 1.29. In addition, the content of bound ferulic acid groups in AX was determined to be 40.03 ± 2.24 μmol/g.

### 2.2. Sample Preparation

MP samples were prepared using 30 g of potato flakes as the base material. AX was supplemented at concentrations of 0.1% and 0.2% (*w*/*w*, based on the dry weight of potato flakes). To ensure uniform dispersion and avoid lump formation, 100 mL of ultrapure water was first added under gentle stirring. Subsequently, another 100 mL of ultrapure water was incorporated, and the mixture was heated in a boiling water bath at 100 °C for 15 min to achieve complete starch gelatinization. Continuous stirring was applied during heating to ensure uniform heat distribution and homogeneous hydration. MP gels without AX supplementation were used as blank controls.

The prepared MP samples were cooled to room temperature (approximately 25 °C), sealed in airtight food-grade containers and stored at 4 °C. Aliquots were collected after 0, 1, 3, and 7 d of storage to determine macroscopic indicators including rheological properties, texture, water distribution, water holding capacity, color and sensory quality. Samples for microstructural and crystallographic analyses were rapidly frozen and freeze-dried at the corresponding storage time points. The freeze-dried samples were carefully fractured to expose cross-sections for microstructure observation by scanning electron microscopy (SEM). Meanwhile, the samples were gently ground and sieved through a 100-mesh sieve for the characterization of long-range crystalline structures and short-range ordered structures. All commercial MP samples were stored at 4 °C together with laboratory-prepared samples. All quality indicators were measured under identical experimental conditions and test parameters to ensure reliable and comparable results.

### 2.3. Rheological Properties

Rheological measurements were performed using a rotational rheometer (MCR 102e, Anton Paar, Graz, Austria) fitted with a 20 mm parallel plate geometry, with the test gap calibrated to 1 mm. All MP samples were equilibrated at 25 °C on the test platform before measurement to ensure uniform temperature distribution. All rheological tests were conducted within the linear viscoelastic region, which was confirmed through preliminary strain sweep tests.

For steady-state shear analysis, the shear rate ranged from 0.1 to 10 s^−1^. For dynamic rheological characterization, amplitude sweep tests were conducted at a constant frequency of 1 Hz with a strain range of 0.01–100% to determine the storage modulus (G′) and loss modulus (G″). Subsequently, frequency sweep tests were carried out at a constant strain of 1% (within the linear viscoelastic region) over a frequency range of 0.1–10 Hz to record the changes in G′ and G″.

### 2.4. Texture Profile Analysis (TPA)

Texture profile analysis (TPA) was performed using a texture analyzer (TA.XT.Plus, Stable Micro Systems Ltd., Godalming, UK) fitted with a P/36R cylindrical probe, according to the method reported by Ren et al. [20] with minor modifications. The pre-test speed, test speed and post-test speed were set to 1.0 mm/s, 1.0 mm/s and 10.0 mm/s, respectively. The trigger force was 5.0 g, the compression strain was 50%, and the holding time was 5 s.

### 2.5. Low-Field Nuclear Magnetic Resonance (LF-NMR)

Low-field nuclear magnetic resonance (LF-NMR) measurement was carried out using an NMI20-040V-I multi-dimensional micro-imaging analyzer (Niumag Electric Corporation, Suzhou, China). The method was slightly modified from that reported by Sun et al. [21]. Briefly, 3.0 g of MP sample was weighed and placed into an NMR tube. The Carr–Purcell–Meiboom–Gill (CPMG) pulse sequence was adopted. The test parameters were set as follows: sampling bandwidth 250 kHz, gain level 1, relaxation decay time 8000 ms, echo time 0.2 ms, 16,000 echoes and 4 scan accumulations. Data inversion was completed via the built-in analysis software of the apparatus.

### 2.6. Water Holding Capacity (WHC)

The water holding capacity (WHC) of MP samples stored at 4 °C for different periods was determined via the centrifugation method. Freshly prepared samples were sealed and kept at 4 °C until reaching the scheduled storage time. After uniform mixing, 2.0 g of homogenized sample was accurately weighed into a pre-weighed centrifuge tube, then centrifuged at 10,000 rpm for 10 min at room temperature. The separated free water was discarded, and the total weight of the centrifuge tube containing residual samples was measured immediately to calculate the WHC value.

$$\mathrm{WHC}\;\left(\%\right)=\frac{W_{2}-W_{0}}{W_{1}-{\;W}_{0}}\;\times \;100\%$$

where *W*_0_ represents the mass of the empty centrifuge tube; *W*_1_ and *W*_2_ are the total masses of the centrifuge tube containing samples before and after centrifugation, respectively.

### 2.7. X-Ray Diffraction (XRD)

X-ray diffraction (XRD) patterns were collected by a TD-3700 X-ray diffractometer (Tongda Technology Co., Ltd., Dandong, China) based on the method reported by Jiang et al. [22]. An appropriate amount of freeze-dried MP powder was uniformly laid on a glass slide. The test was conducted with Cu-Kα radiation under operating conditions of 40 kV and 40 mA. The diffraction scanning range was set from 5° to 45°, with a scanning rate of 1°/min and a step size of 0.02°.

### 2.8. Fourier Transform Infrared Spectroscopy (FTIR)

Fourier transform infrared (FTIR) spectra were acquired using a Spectrum 100 FTIR spectrometer (PerkinElmer, Waltham, MA, USA). The freeze-dried MP powder was blended with potassium bromide at a mass ratio of 1:100 (*w*/*w*), then fully ground and compressed into pellets for testing. The scanning wavenumber range was set from 4000 to 400 cm^−1^.

### 2.9. Scanning Electron Microscopy (SEM)

Scanning electron microscopy (SEM) observation was conducted with a Sigma 360 scanning electron microscope (Zeiss, Oberkochen, Germany). Freeze-dried MP samples were fractured in liquid nitrogen, sputter-coated with gold, and then photographed under an accelerating voltage of 10 kV.

### 2.10. Chromaticity Values

Chromaticity parameters, including L*, a*, and b* of MP samples at different storage periods, were determined by an NH310 colorimeter (3nh Co., Ltd., Shenzhen, China). The instrument was calibrated using a standard white plate prior to measurement.

### 2.11. Sensory Quality

A sensory evaluation panel composed of 10 trained food science assessors (5 males and 5 females, aged 20–30 years) was established to assess the color, odor, texture and mouthfeel of MP samples.

All panelists received unified pre-experiment training to fully clarify the definition of each evaluation index and scoring standard, and were well acquainted with evaluation rules and operating procedures. All samples were randomly sorted and marked with three-digit random codes. Panelists rinsed their mouths with purified water before assessment and between sample tests to avoid residual flavor interference.

Sensory evaluation was carried out in a standard sensory evaluation room with independent evaluation cubicles under uniform white light at 25 ± 2 °C, free from noise and extraneous odors. The detailed scoring standards are presented in Table S1.

### 2.12. Quality Comparison with Commercial Products

Laboratory-prepared MP samples were compared with three commercial MP products in terms of overall quality. Their texture characteristics, chromaticity parameters and sensory attributes were measured following the identical methods mentioned above.

### 2.13. Statistical Analysis

All experiments except XRD were conducted in triplicate, and the results were expressed as mean ± standard deviation (*n* = 3). Sensory evaluation was performed with ten trained panelists. Statistical analyses were carried out using IBM SPSS Statistics 27. One-way analysis of variance (ANOVA) combined with Duncan’s multiple range test was used to evaluate significant differences at *p* < 0.05. All figures were plotted using Origin 2025 software.

## 3. Results and Discussions

### 3.1. Rheological Properties

**Figure 1:**
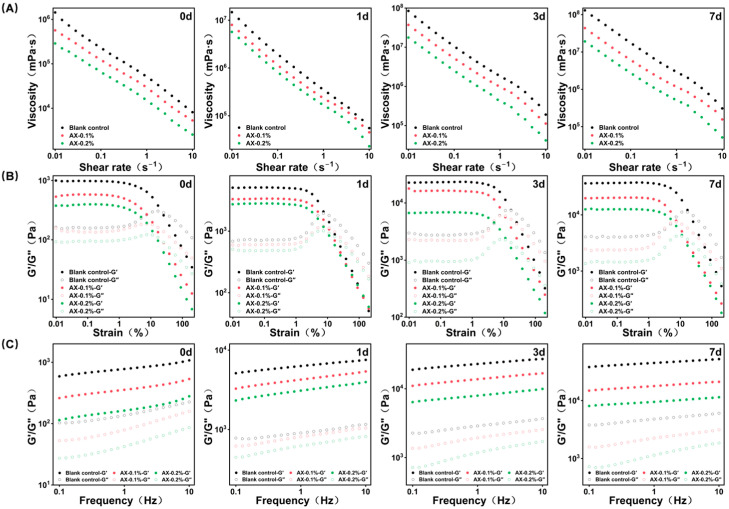
(**A**) Steady shear, (**B**) amplitude sweep, and (**C**) frequency sweep of MP with or without AX at 0.1% and 0.2% during storage at 4 °C for 0, 1, 3, and 7 d.

Rheological properties are important indicators reflecting the internal structural stability and microstructural evolution of starch-based gels. To investigate the regulatory effect of AX on the texture and retrogradation behavior of MP during refrigerated storage at 4 °C, the steady shear and dynamic viscoelastic properties of samples were systematically analyzed. As presented in Figure 1A, the incorporation of AX at 0.1% and 0.2% both lowered the initial apparent viscosity of the MP at 0 d, and the reduction effect was positively related to the addition level. At a shear rate of 0.01 s^−1^, the apparent viscosities of the blank control, AX-0.1%, and AX-0.2% groups were 1.43 × 10^6^, 5.67 × 10^5^, and 2.88 × 10^5^ mPa·s, respectively. This result suggested that AX weakened the intermolecular interactions within the gel network and enhanced the fluidity of the system, which corresponded to a softer and more deformable texture at the macroscopic level [23]. During storage up to 7 d, the apparent viscosity of all samples increased substantially due to the continuous starch retrogradation. Nevertheless, compared with the blank control, AX supplementation restricted the elevation of apparent viscosity (Figure 1A). At 7 d, the apparent viscosity of the blank control rose to approximately 1.30 × 10^8^ mPa·s, whereas those of the AX-0.1% and AX-0.2% groups were only 4.36 × 10^7^ and 1.92 × 10^7^ mPa·s, respectively (Figure 1A). These results demonstrated that AX softened the fresh MP matrix and helped alleviate excessive structural hardening during storage.

To further investigate the regulatory effect of AX on the structural stability and dynamic viscoelasticity of MP gels during refrigerated storage at 4 °C, amplitude and frequency sweep tests were carried out within the predetermined linear viscoelastic region (Figure 1B,C). The rheological properties of all samples showed continuous reinforcement of the gel network induced by starch retrogradation from 0 to 7 d. With the prolongation of storage time, the storage modulus (G′) of all groups increased, and the linear viscoelastic region (LVR) was extended, corresponding to a higher yield strain. This result indicated that the MP gradually lost its desired soft, creamy, and melt-in-the-mouth texture during storage. The incorporation of AX into MP inhibited this excessive gel hardening phenomenon. As displayed in Figure 1B, the G′ and G″ values as well as the LVR of AX-supplemented groups were consistently lower than those of the blank control during 0–7 d. At 0 d, the initial G′ values at 0.01% strain for the blank control, AX-0.1%, and AX-0.2% groups were 996.72, 532.63, and 375.56 Pa, respectively. Meanwhile, MP samples with AX yielded at a lower strain point (approximately 11% for AX-0.1% and AX-0.2% groups). This result demonstrated that AX reduced the initial viscoelasticity, enabling the system to undergo smoother deformation. Furthermore, the G′ value of the blank control increased greatly to 3.67 × 10^4^ Pa after 7 d, accompanied by a broadened LVR. In comparison, the G′ value of the AX-0.2% group was restricted to only 1.26 × 10^4^ Pa, and its LVR remained relatively narrow (Figure 1B). Overall, these results indicated that AX attenuated starch retrogradation and helped maintain gel strength at a lower and more desirable level. The steric hindrance induced by AX may interfere with the ordered double-helix formation and dense physical cross-linking of starch molecules, thus helping preserve the structural homogeneity and paste-like flexibility of the MP network during cold storage [24].

### 3.2. Texture Profile Analysis (TPA)

The texture properties of MP were evaluated via TPA to quantify the macroscopic sensory attributes corresponding to the rheological changes during storage (Table 1). Hardness, the core indicator characterizing the softness of MP, was affected by AX incorporation. The hardness of the blank control increased from 107.11 ± 5.97 g to 520.74 ± 10.72 g over 7 d, indicating substantial hardening during storage. AX at 0.2% exerted a softening effect, with its hardness remaining at a low level of 214.32 ± 5.13 g after 7 d (Table 1). Consistent with the apparent viscosity and G′ results above, AX incorporation reduced the initial hardness of MP and markedly retarded the stiffening process during refrigerated storage.

Springiness, cohesiveness and resilience are key parameters reflecting the structural integrity, deformation recovery capacity and smooth mouthfeel of the gel network [25]. With the prolongation of refrigerated storage, these three indices changed. For instance, the cohesiveness of the blank control decreased from 0.94 ± 0.01 to 0.33 ± 0.01, which suggested that the highly retrograded starch matrix became increasingly brittle, loose and prone to structural fracture (Table 1). AX-supplemented groups showed good retention of these attributes throughout the storage period. At 7 d, the springiness, cohesiveness and resilience of the AX-0.2% group were higher than those of the blank control. These results demonstrated that AX helped maintain the structural integrity of MP and preserve the flexibility of the gel network. These TPA results indicated that AX attenuated starch retrogradation, thus avoiding the transformation of MP into a brittle and crumbly solid and maintaining its desirable paste-like texture [26].

### 3.3. Low-Field Nuclear Magnetic Resonance (LF-NMR) and Water Holding Capacity (WHC)

**Figure 2:**
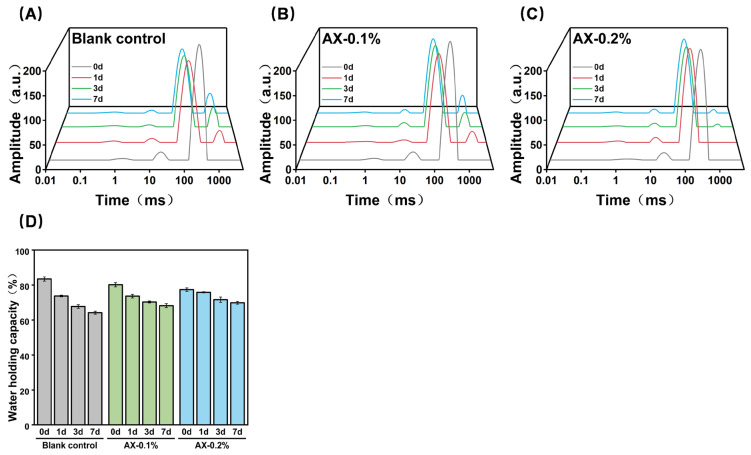
(**A**–**C**) Water distribution and (**D**) water holding capacity (WHC) of MP with or without AX during storage at 4 °C for 0, 1, 3, and 7 d.

Water state and migration behavior are critical factors governing syneresis, microstructural stability, and macroscopic sensory quality of MP. LF-NMR was employed to characterize water distribution and mobility by measuring transverse relaxation times (*T*_2_). Generally, *T*_21_, *T*_22_, *T*_23_, and *T*_24_ represent strongly bound water, semi-bound water, immobilized water, and free water, respectively, which could indicate progressively enhanced molecular mobility of samples. Additionally, *A*_2_ denotes the relative peak area proportion of each water fraction [27]. The dynamic moisture transitions of MP samples during refrigerated storage are presented in Figure 2 and Table 2. At 0 d, the relaxation times (i.e., *T*_21_, *T*_22_ and *T*_23_) of the AX-supplemented groups were higher than those of the blank control. This indicated that AX slightly increased the freedom of water molecules, likely due to its strong hydrophilicity, which was correlated with the softer texture and lower G′ values observed in the TPA and rheological tests as described above (Figure 1 and Table 1).

In addition, the *T*_21_, *T*_22_ and *T*_23_ values of all samples progressively decreased during 7 d refrigerated storage, which suggested restricted water molecular mobility in MP samples (Figure 2 and Table 2). This finding was in accordance with a previous study reporting that water binding is notably altered as starch molecular chains are rearranged during starch retrogradation [28]. However, the phase distribution of water fractions (*A*_2_ values) revealed a distinct difference in network stability among samples. For instance, the immobilized water proportion (*A*_23_) of the blank control decreased from 92.95 ± 0.14% to 81.91 ± 1.03%, while the *T*_24_ peak (representing highly mobile free water expelled from the network) was observed at 1 d (Figure 2A), and the *A*_24_ values increased to 14.03 ± 0.91% by 7 d (Figure 2A and Table 2). The extensive moisture migration from *A*_23_ to *A*_24_ may explain the macroscopic syneresis, brittleness, and loss of cohesiveness quantified in the earlier TPA results [29] (Table 1). In contrast, AX helped retard water migration, as evidenced by a relatively milder reduction in *T*_2_ values throughout the storage period (Figure 2B,C, Table 2). The immobilized water fraction (i.e., *A*_23_ value) of the AX-0.2% group remained stable compared with the blank control and maintained a high level of 94.74 ± 0.58% after 7 d. Moreover, the appearance of the free water peak (i.e., *T*_24_ peak) was delayed to 3 d (Figure 2C), and its final proportion (*A*_24_) was reduced to 1.65 ± 0.14% (Figure 2C and Table 2).

The variations in WHC of MP samples during refrigerated storage further supported the water regulation effect of AX (Figure 2D and Table 2). The WHC values of all samples continuously decreased with increasing storage time. The blank control group exhibited the sharpest decline, with WHC dropping from 83.44 ± 1.23% at 0 d to 64.14 ± 0.83% at 7 d, revealing impaired water retention capacity. Although the initial WHC of AX-supplemented groups was slightly lower than that of the blank control group, their decline magnitudes were narrower. After 7 d of storage, the WHC of the AX-0.1% group and the AX-0.2% group remained at 68.21 ± 1.14% and 69.86 ± 0.76%, respectively, which were higher than those of the blank control group.

These results suggest that AX acts as a moisture stabilizer in starch-based food. Previous studies have reported that AX retards water migration and improves the water-holding capacity and syneresis resistance of MP [30]. Overall, these LF-NMR results combined with macroscopic WHC data provide microstructural evidence that AX softens the initial texture and stabilizes the gel network to reduce syneresis during storage [31]. This highlights the functionality of AX in texture regulation and shelf-life extension of cold-chain MP from a water dynamics perspective.

### 3.4. X-Ray Diffraction (XRD)

**Figure 3:**
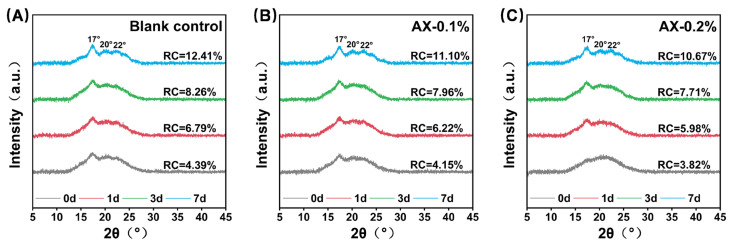
(**A**–**C**) X-ray diffraction (XRD) patterns and relative crystallinity (RC) of MP with or without AX during refrigerated storage at 4 °C for 0, 1, 3, and 7 d.

Starch recrystallization serves as the core molecular factor inducing rheological stiffening, texture brittleness and water syneresis of MP during refrigerated storage [32]. XRD measurement was performed to characterize variations in starch crystal structure and relative crystallinity (RC), aiming to explore the anti-retrogradation property of AX at the molecular scale. As shown in Figure 3A,B, a weak diffraction peak appeared at approximately 2*θ* = 17° in the blank control and AX-0.1% samples, indicating rapid formation of crystal structure and initial retrogradation immediately after sample preparation and cooling. This peak was barely detectable in AX-0.2% sample with a lower RC value of 3.82%, indicating that high-content AX helped restrain the association of starch molecular chains in the early storage stage (Figure 3C).

Furthermore, the intensity of the diffraction peak at approximately 2*θ* = 17° rose gradually in all samples as storage time prolonged. By the later storage period (i.e., after 3 and 7 d), new characteristic retrogradation peaks emerged at approximately 2*θ* = 20° and 22° (Figure 3), matching the formation of typical B-type and V-type crystallites [33]. Specifically, the RC value of the blank control rose about 2.8 times, growing from 4.39% at 0 d to 12.41% at 7 d. This finding is consistent with the foregoing rheological and TPA results. Moreover, the addition of AX inhibited starch recrystallization. Lower RC values were observed in AX-supplemented samples compared with the blank control during the whole refrigerated storage period. For example, the RC values of AX-0.1% and AX-0.2% samples reached 11.10% and 10.67% at 7 d (Figure 3), revealing that AX may interfere with the ordered crystalline arrangement of starch in MP. Existing studies have shown that AX may interact with starch chains, thereby hindering the alignment and aggregation of amylopectin double helices [34]. In addition, AX weakens the initial continuous gel network and physically isolates starch chains, which may disrupt the favorable microenvironment for crystal growth. Hence, the decline of RC values explains why AX maintains pasty flexibility and improves storage stability of MP during refrigerated storage.

### 3.5. Fourier Transform Infrared Spectroscopy (FTIR)

**Figure 4:**
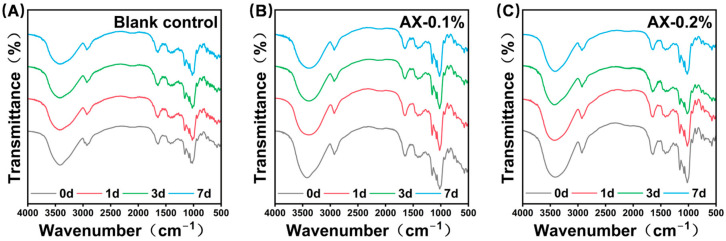
(**A**–**C**) Fourier transform infrared (FTIR) spectra of MP with or without AX during refrigerated storage at 4 °C for 0, 1, 3, and 7 d.

Starch short-range ordered structure represents the local arrangement regularity of starch molecular chains, predominantly amylose, and acts as the basic foundation for forming long-range crystalline structures. FTIR measurement was adopted to monitor variations in starch short-range ordered structures via analyzing functional group vibration features. In detail, the absorbance ratio R_1047/1022_ indicates starch short-range order degree, and R_995/1022_ stands for the relative content of double helix and amorphous regions inside starch molecules [35]. The FTIR spectra of MP samples during storage are presented in Figure 4.

As listed in Table 3, the short-range order degree of the blank control sample increased during refrigerated storage. The absorbance ratio R_1047/1022_ increased from 1.003 ± 0.001 to 1.034 ± 0.001 (increased by 3.1%), and the ratio R_995/1022_ rose from 1.015 ± 0.002 to 1.041 ± 0.002 (increased by 2.6%). These results indicated that starch molecular chains continuously rearranged and aggregated during storage, accompanied by the gradual formation of double helix structures and the ordering of amorphous regions. This variation was consistent with the increasing trend of RC in XRD analysis (Figure 3), further supporting that starch retrogradation was the key factor causing quality deterioration of MP during refrigerated storage.

At the same storage time, both R_1047/1022_ and R_995/1022_ of AX-supplemented samples were higher than those of the blank control sample in a dose-dependent manner. Although AX-supplemented samples showed higher R_1047/1022_ values than the blank control, this difference likely reflects additional short-range ordering induced by AX–starch interactions, rather than retrogradation per se, which is consistent with previous reports that exogenous polysaccharides can form new ordered structures with starch [36,37].

The increased extent of short-range ordered structure can reflect the inhibitory effect of AX on starch retrogradation. After 7 d of refrigerated storage, the increments of R_1047/1022_ and R_995/1022_ in the 0.1% AX-supplemented sample were only 2.2% and 1.7%, respectively, while those in the 0.2% AX-supplemented sample were both 1.1%, which were lower than those in the blank control sample. Due to its high branching degree and good water solubility, AX may interact with starch molecular chains, thereby inhibiting the spontaneous rearrangement and aggregation of starch molecules [38]. In addition, AX weakened the initial gel network of MP and restricted the mobility of starch chains, thereby inhibiting the transformation from short-range ordered structures to long-range crystalline structures. AX delayed the retrogradation-related quality deterioration of MP at the molecular level and enhanced the storage stability of samples.

### 3.6. Scanning Electron Microscopy (SEM)

**Figure 5:**
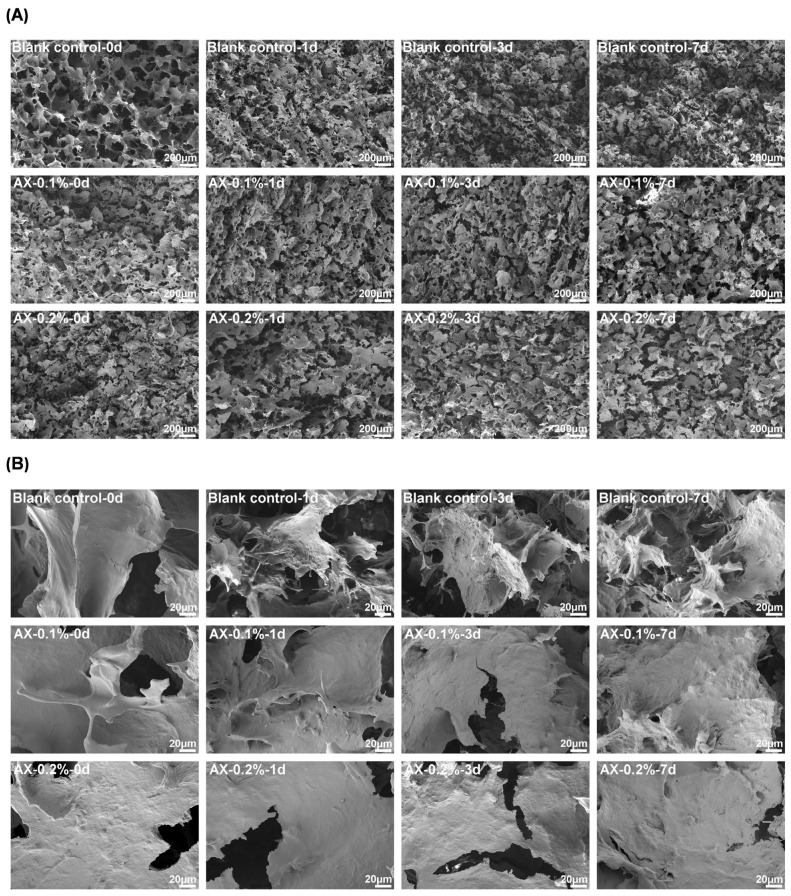
Scanning electron microscopy (SEM) images of MP with or without AX during refrigerated storage at 4 °C for 0, 1, 3, and 7 d. (**A**) 50× magnification (scale bar: 200 μm); (**B**) 500× magnification (scale bar: 20 μm).

The changes in the microscopic morphology of MP during refrigerated storage are presented in Figure 5. At 0 d, the blank control group showed a porous honeycomb structure with varying pore sizes, uniform distribution, a continuous gel network, and thick pore walls with surface wrinkles. In contrast, AX-0.1% and AX-0.2% samples exhibited a looser microstructure without a well-defined honeycomb structure. The gel networks of AX-supplemented MP samples were interrupted, with thinner pore walls compared with the blank control.

During refrigerated storage up to 7 d, the microstructures of all MP samples changed, with pore shrinkage, pore wall roughening, and gradual collapse of the gel network. In the blank control, the honeycomb network gradually collapsed from 1 d to 7 d, forming a compacted structure with fibrous protrusions, accumulated wrinkles, and reduced porosity. This structural shrinkage coincided with the increase in free water (*T*_24_) observed by LF-NMR (Figure 2A–C and Table 2).

Compared with the blank control, AX-supplemented groups showed less structural collapse. The AX-0.1% group displayed some pore roughening, but the extent of change was limited. The AX-0.2% group exhibited less pore shrinkage, thinner pore walls, and fewer surface irregularities compared with the blank control. These observations suggest that AX may reduce the extent of network compaction during refrigerated storage.

### 3.7. Chromaticity Values

**Figure 6:**
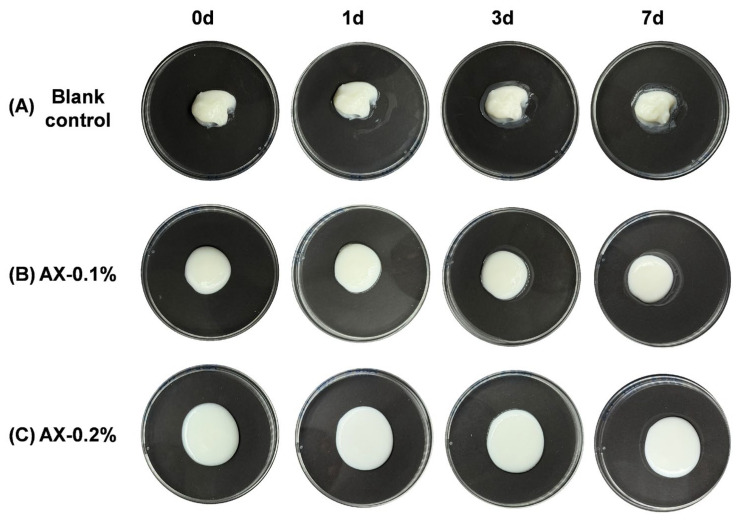
(**A**–**C**) Visual appearances of MP with or without AX during refrigerated storage at 4 °C for 0, 1, 3, and 7 d.

Color changes of MP during refrigerated storage are shown in Table 4 and Figure 6.

The L* value of the blank control group decreased by 17.9% after 7 d of refrigerated storage, and the MP surface turned darker with reduced gloss (Figure 6A). This decrease could be ascribed to enhanced light absorption caused by starch retrogradation, as well as aggravated lipid oxidation during storage [39]. The a* value remained negative (greenish hue), and its absolute value declined with extended storage, indicating a gradual shift toward a neutral color tone. The b* value increased by up to 140.3% with yellowing, which was closely associated with structural rearrangement induced by starch retrogradation [39].

The initial L* value of the AX groups was lower than that of the blank control group, with more negative a* values and lower b* values, indicating that AX endowed MP with an initial color that was slightly darker, more greenish, and lower yellowness, which may be related to the inherent color characteristics of AX (Figure 6B,C). The L* value of the AX-0.1% group decreased by 13.7% at 7 d of refrigerated storage, while that of the AX-0.2% group decreased by only 9.5%, demonstrating that AX may delay the darkening of MP. The a* values of the AX groups remained more negative (stronger green hue) at all refrigerated storage time points with a mild variation range, suggesting that AX may inhibit the shift of green hue toward a neutral hue. The absolute increment of the b* value was 12.16 for the AX-0.1% group and 11.16 for the AX-0.2% group, both lower than 14.56 for the blank control group. Although the relative increment of the b* value (167.1%) in the AX-0.2% group was higher than that in the blank control group, its absolute b* value was lower than those in the blank control group and the AX-0.1% group at all refrigerated storage time points, indicating that 0.2% AX may exert an inhibitory effect on yellowing. This might be associated with the antioxidant activity of feruloyl groups in AX molecules, as the content of feruloyl groups was higher in the system supplemented with 0.2% AX [40].

### 3.8. Sensory Quality

Sensory quality evaluation results are shown in Table 5. At 0 d, there was no difference in the total sensory scores among the three groups of MP. With prolonged refrigerated storage, the total sensory scores of the blank control group and AX-0.1% group decreased, which may be attributed to starch retrogradation. The total sensory score of the AX-0.2% group at 1 d of refrigerated storage was higher than that at 0 d, which might be related to its initially soft texture. The moderate recovery of hardness after short-term refrigerated storage may contribute to a corresponding improvement in sensory acceptability. The total score of the blank control group decreased by 24.8% after 7 d of refrigerated storage, indicating deterioration of sensory quality; the decrease was 21.1% for the AX-0.1% group and as low as 12.2% for the AX-0.2% group (calculated based on the initial score at 0 d). From 1 d of refrigerated storage onward, the total sensory scores of the AX groups were higher than those of the blank control group, and the AX-0.2% group maintained the highest scores from 1 to 7 d of refrigerated storage. This demonstrated that AX may delay the deterioration of sensory quality of MP, with the effect at the addition level of 0.2%.

AX may exert a preservation effect on all sensory attribute scores. Except that the texture score of the AX-0.2% group at 0 d was slightly lower than that of the blank control group, the sensory attribute scores of the AX groups at other refrigerated storage time points were all higher than those of the blank control group at the corresponding time points. Although some data did not reach a statistically significant difference, the observed numerical trends still suggested that AX may exert an effect on improving the sensory acceptability of MP during refrigerated storage.

### 3.9. Quality Comparison with Commercial Products

To evaluate the commercial application potential of AX-supplemented MP, three types of mainstream commercial instant MP including both pure potato-based and lightly seasoned variants were selected for quality comparison under uniform storage and testing conditions. All commercial samples were verified via product labels to contain no exogenous stabilizers, thickeners or hydrocolloids, which ensured the formula system was comparable to that of the laboratory-prepared samples. Considering that the precise raw material ratios and industrial processing parameters of commercial products are undisclosed, this comparison was performed to preliminarily assess the comprehensive quality and edible advantages of laboratory-prepared samples, rather than to conduct a rigorous mechanistic comparison.

Most commercial MP products made from potato flour are reconstitutable products, which requires consumers to rehydrate before consumption, resulting in inconsistent texture and rendering objective quality unfeasible. Therefore, this study selected commercial whole-potato-based MP (ready-to-eat without further preparation) as the control group for quality comparison. The results of the quality comparison are presented in Tables S2–S4.

The hardness of the blank control group and AX groups was lower than that of commercial products, and the hardness of the AX-0.2% group was only 6.4% of that of commercial product 2. Except for the higher springiness of commercial product 3, there was no difference in springiness among other samples, indicating that the softening effect of AX did not reduce the deformation recovery ability. The laboratory-prepared samples exhibited higher cohesiveness with a more uniform and delicate microstructure. The changes in gumminess and chewiness were consistent with those of hardness; the corresponding values in the AX groups were lower, especially the AX-0.2% group, which met the quality requirements of smooth and melt-in-the-mouth texture. In contrast, the commercial products exhibited variations in quality attributes. The resilience of the AX groups was slightly lower, but the difference was small and had no obvious effect on the overall quality.

The L* value of the blank control group was the highest, and that of the AX groups was slightly lower but fell within the value range of commercial products. The a* values of the AX groups were more negative (greener hue), and the AX-0.2% group was close to commercial product 2. The difference in b* values was the largest: the b* values of laboratory-prepared samples (7.48 ± 0.24 to 11.50 ± 0.17) were lower than those of commercial products (28.10 ± 0.47 to 43.95 ± 0.05), exhibiting a lighter color. The commercial products exhibited a strong yellow hue, which may be related to their raw materials and processing technology. MP supplemented with AX had similar lightness, a stronger green hue and lower yellow hue compared with commercial products, exhibiting a fresh and light color close to freshly prepared samples, which may be preferred by consumer demand for natural and fresh ready-to-eat foods.

There was no difference in the total scores between the three laboratory-prepared groups and commercial products, and the sensory acceptability of the AX groups was comparable to that of commercial products, among which the AX-0.1% group had the highest total score. The color score of commercial products was higher, which may be related to the differences in raw materials and processing technology. There was no difference in odor score among all groups, indicating that AX did not introduce any off-flavor. The laboratory-prepared groups exhibited higher scores in texture and mouthfeel, which was consistent with the higher cohesiveness and lower hardness in texture analysis results, showing higher fineness and smoothness. In conclusion, the sensory level of the AX-supplemented groups was comparable to that of commercial products, with better core texture indicators, suggesting good commercial application potential.

## 4. Conclusions

This study investigated the effects of AX on the quality and starch retrogradation of MP during refrigerated storage at 4 °C for 7 d. The results demonstrated that starch retrogradation was a key factor leading to the increased hardness, water exudation, structural deterioration, color degradation and sensory quality reduction of MP during refrigerated storage. AX may inhibit the ordered arrangement and recrystallization of starch molecules, thereby retarding starch retrogradation, and the improvement effect was positively correlated with the AX addition level. AX supplementation reduced the initial rheological modulus and hardness of MP, inhibited texture hardening during storage, and maintained color and sensory quality stability. At the microscopic level, AX may immobilize water in MP, reduce free water formation, inhibit the formation of crystal structures, and maintain microstructure integrity. Quality comparison with commercial MP showed that laboratory-prepared AX-supplemented MP exhibited similar sensory quality to commercial products, with a softer and finer texture. Unlike conventional hydrocolloids that inhibit retrogradation by increasing viscosity and strengthening the gel network, AX modulates starch retrogradation while preserving the ideal soft and smooth mouthfeel of MP, highlighting its advantages in ready-to-eat starchy foods.

Although the sensory panel in this study consisted of 10 trained members with a modest sample size, it should be clarified that the purpose of sensory evaluation was not to compare superiority or preference among samples, but to verify whether AX supplementation negatively affected the sensory quality of MP. A trained panel of 10 members was adequate to identify differences among samples, which fulfilled the core objectives of this study.

Furthermore, the optimal shelf life of commercial fresh ready-to-eat MP during refrigerated storage is generally within 7 d. Hence, the 7 d refrigerated storage selected in this study is adequate to fully characterize texture deterioration and starch retrogradation of MP, reflect quality changes during the actual optimal consumption period, and validate the regulatory effect of AX.

In conclusion, as a natural functional polysaccharide, AX may inhibit starch retrogradation and enhance the storage stability of MP. This study improves the application potential of AX in ready-to-eat starchy foods and provides a novel strategy for quality improvement of ready-to-eat potato products.

## Figures and Tables

**Table 1:** Texture profile analysis of MP with or without AX at 0.1% and 0.2% during storage at 4 °C for 0, 1, 3, and 7 d.

	Storage Time	Hardness (g)	Springiness	Cohesiveness	Gumminess	Chewiness	Resilience
Blank control	0 d	107.11 ± 5.97 ^d^	0.97 ± 0.00 ^a^	0.94 ± 0.01 ^a^	100.30 ± 4.54 ^d^	97.72 ± 4.18 ^a^	0.18 ± 0.01 ^a^
	1 d	296.32 ± 8.27 ^c^	0.74 ± 0.03 ^b^	0.40 ± 0.01 ^b^	119.16 ± 5.53 ^c^	88.33 ± 4.28 ^a^	0.10 ± 0.00 ^b^
	3 d	417.67 ± 9.59 ^b^	0.56 ± 0.01 ^c^	0.37 ± 0.00 ^c^	152.84 ± 4.87 ^b^	84.87 ± 4.63 ^b^	0.07 ± 0.00 ^c^
	7 d	520.74 ± 10.72 ^a^	0.34 ± 0.02 ^d^	0.33 ± 0.01 ^d^	173.30 ± 9.79 ^a^	58.79 ± 6.46 ^c^	0.04 ± 0.00 ^d^
AX-0.1%	0 d	61.11 ± 1.53 ^d^*	0.97 ± 0.00 ^a^	0.90 ± 0.01 ^a^*	55.07 ± 1.77 ^b^*	53.15 ± 1.53 ^c^*	0.17 ± 0.01 ^a^*
	1 d	184.46 ± 14.17 ^c^*	0.84 ± 0.04 ^b^*	0.56 ± 0.01 ^b^*	103.81 ± 6.81 ^a^*	87.25 ± 5.10 ^a^	0.11 ± 0.01 ^b^*
	3 d	261.48 ± 13.10 ^b^*	0.72 ± 0.01 ^c^*	0.38 ± 0.02 ^c^	98.77 ± 1.84 ^a^*	71.17 ± 2.01 ^b^*	0.07 ± 0.00 ^c^
	7 d	315.07 ± 9.44 ^a^*	0.56 ± 0.02 ^d^*	0.35 ± 0.00 ^d^	104.56 ± 7.60 ^a^*	58.97 ± 6.21 ^c^	0.05 ± 0.00 ^d^*
AX-0.2%	0 d	42.59 ± 2.82 ^d^*	0.96 ± 0.01 ^a^	0.88 ± 0.01 ^a^*	37.27 ± 2.80 ^c^*	35.72 ± 3.01 ^c^*	0.16 ± 0.01 ^a^*
	1 d	88.18 ± 2.38 ^c^*	0.81 ± 0.02 ^b^*	0.64 ± 0.01 ^b^*	56.19 ± 1.78 ^b^*	45.54 ± 1.98 ^b^	0.13 ± 0.01 ^b^*
	3 d	117.78 ± 8.23 ^b^*	0.74 ± 0.01 ^c^*	0.49 ± 0.01 ^c^*	58.21 ± 4.66 ^b^*	43.05 ± 3.21 ^b^*	0.11 ± 0.01 ^c^*
	7 d	214.32 ± 5.13 ^a^*	0.62 ± 0.00 ^d^*	0.41 ± 0.01 ^d^*	86.86 ± 1.13 ^a^*	53.66 ± 0.82 ^a^	0.08 ± 0.01 ^d^*

Note: Data are expressed as mean ± standard deviation; different lowercase letters indicate significant differences among different storage times within the same group (*p* < 0.05); * indicates significant difference compared with the blank control group at the same storage time (*p* < 0.05).

**Table 2:** Water distribution parameters and water holding capacity (WHC) of MP with or without AX during storage at 4 °C for 0, 1, 3, and 7 d.

	Storage Time	*T*_21_ (ms)	*T*_22_ (ms)	*T*_23_ (ms)	*T*_24_ (ms)	*A*_21_ (%)	*A*_22_ (%)	*A*_23_ (%)	*A*_24_ (%)	WHC (%)
Blank control	0 d	1.56 ± 0.19 ^a^	21.55 ± 0.43 ^a^	292.53 ± 0.00 ^a^	ND	1.50 ± 0.09 ^c^	5.55 ± 0.21 ^a^	92.95 ± 0.14 ^a^	ND	83.44 ± 1.23 ^a^
	1 d	0.67 ± 0.05 ^b^	11.18 ± 0.82 ^b^	155.07 ± 3.08 ^b^	1489.13 ± 0.00 ^a^	1.65 ± 0.17 ^bc^	2.82 ± 0.11 ^b^	88.73 ± 0.22 ^b^	6.80 ± 0.13 ^b^	73.69 ± 0.47 ^b^
	3 d	0.60 ± 0.48 ^b^	10.00 ± 1.26 ^bc^	127.48 ± 4.41 ^c^	1210.26 ± 41.90 ^b^	2.17 ± 0.07 ^a^	2.51 ± 1.10 ^b^	82.60 ± 0.34 ^c^	12.73 ± 1.02 ^a^	67.77 ± 1.03 ^c^
	7 d	0.47 ± 0.06 ^b^	9.04 ± 1.37 ^c^	116.20 ± 2.34 ^d^	1128.84 ± 0.00 ^c^	2.00 ± 0.32 ^ab^	2.06 ± 0.28 ^b^	81.91 ± 1.03 ^c^	14.03 ± 0.91 ^a^	64.14 ± 0.83 ^d^
AX-0.1%	0 d	1.70 ± 0.03 ^a^	23.64 ± 0.93 ^a^*	302.83 ± 0.00 ^a^*	ND	1.36 ± 0.24 ^a^	5.38 ± 0.07 ^a^	93.26 ± 0.29 ^a^	ND	80.18 ± 1.21 ^a^*
	1 d	0.81 ± 0.03 ^b^*	12.82 ± 0.26 ^b^*	156.85 ± 0.00 ^b^	1750.51 ± 34.79 ^a^*	1.44 ± 0.13 ^a^	1.93 ± 0.14 ^b^*	90.69 ± 0.27 ^b^*	5.94 ± 0.06 ^c^*	73.62 ± 1.03 ^b^
	3 d	0.65 ± 0.01 ^c^	11.18 ± 0.82 ^c^*	136.57 ± 0.00 ^c^*	1411.55 ± 161.29 ^b^	1.45 ± 0.29 ^a^*	2.06 ± 0.52 ^b^	88.34 ± 0.48 ^c^	8.14 ± 0.27 ^b^*	70.30 ± 0.56 ^c^*
	7 d	0.48 ± 0.12 ^d^	10.94 ± 1.02 ^c^	124.54 ± 2.50 ^d^*	1357.97 ± 27.30 ^b^*	1.32 ± 0.38 ^a^*	1.97 ± 0.14 ^b^	86.99 ± 0.78 ^d^*	9.72 ± 1.03 ^a^*	68.21 ± 1.14 ^d^*
AX-0.2%	0 d	1.84 ± 0.04 ^a^*	24.46 ± 0.49 ^a^*	309.95 ± 6.16 ^a^*	ND	1.23 ± 0.11 ^b^	4.99 ± 0.12 ^a^*	93.78 ± 0.06 ^b^*	ND	77.41 ± 0.97 ^a^*
	1 d	0.86 ± 0.02 ^b^*	13.27 ± 0.26 ^b^*	160.54 ± 3.19 ^b^	ND	1.62 ± 0.17 ^a^	3.01 ± 0.12 ^b^	95.37 ± 0.07 ^a^*	ND	75.87 ± 0.33 ^a^*
	3 d	0.69 ± 0.01 ^c^	11.96 ± 0.24 ^c^*	138.17 ± 2.78 ^c^*	1507.21 ± 59.55 ^a^*	1.40 ± 0.20 ^ab^*	2.44 ± 0.01 ^c^	94.87 ± 0.24 ^a^*	1.28 ± 0.04 ^b^*	71.61 ± 1.49 ^b^*
	7 d	0.51 ± 0.03 ^d^	11.17 ± 0.23 ^d^*	126.79 ± 1.11 ^d^*	1424.57 ± 30.60 ^a^*	1.28 ± 0.09 ^b^*	2.33 ± 0.40 ^c^	94.74 ± 0.58 ^a^*	1.65 ± 0.14 ^a^*	69.86 ± 0.76 ^b^*

Note: Data are expressed as mean ± standard deviation; different lowercase letters indicate significant differences among different storage times within the same group (*p* < 0.05); * indicates significant difference compared with the blank control group at the same storage time (*p* < 0.05); ND: Not detected.

**Table 3:** Fourier transform infrared (FTIR) absorbance ratios of MP with or without AX during refrigerated storage at 4 °C for 0, 1, 3, and 7 d.

	Storage Time	R_1047/1022_	R_995/1022_
Blank control	0 d	1.003 ± 0.001 ^d^	1.015 ± 0.002 ^d^
	1 d	1.018 ± 0.001 ^c^	1.024 ± 0.002 ^c^
	3 d	1.025 ± 0.002 ^b^	1.032 ± 0.001 ^b^
	7 d	1.034 ± 0.002 ^a^	1.041 ± 0.002 ^a^
AX-0.1%	0 d	1.027 ± 0.002 ^d^*	1.033 ± 0.000 ^c^*
	1 d	1.036 ± 0.001 ^c^*	1.039 ± 0.002 ^bc^*
	3 d	1.041 ± 0.002 ^b^*	1.044 ± 0.003 ^b^*
	7 d	1.050 ± 0.001 ^a^*	1.051 ± 0.006 ^a^*
AX-0.2%	0 d	1.055 ± 0.005 ^b^*	1.058 ± 0.002 ^c^*
	1 d	1.060 ± 0.001 ^ab^*	1.062 ± 0.002 ^bc^*
	3 d	1.063 ± 0.003 ^b^*	1.065 ± 0.003 ^b^*
	7 d	1.067 ± 0.004 ^a^*	1.070 ± 0.002 ^a^*

Note: Data are expressed as mean ± standard deviation; different lowercase letters indicate significant differences among different storage times within the same group (*p* < 0.05); * indicates significant difference compared with the blank control group at the same storage time (*p* < 0.05).

**Table 4:** Color parameters (L*, a*, b*) of MP with or without AX during refrigerated storage at 4 °C for 0, 1, 3, and 7 d.

	Storage Time	L*	a*	b*
Blank control	0 d	89.50 ± 0.03 ^a^	−2.38 ± 0.02 ^d^	10.38 ± 0.02 ^d^
	1 d	82.43 ± 0.12 ^b^	−1.85 ± 0.02 ^c^	15.22 ± 0.03 ^c^
	3 d	76.36 ± 0.02 ^c^	−1.35 ± 0.03 ^b^	19.77 ± 0.01 ^b^
	7 d	73.46 ± 0.03 ^d^	−1.07 ± 0.02 ^a^	24.94 ± 0.02 ^a^
AX-0.1%	0 d	88.48 ± 0.21 ^a^*	−3.51 ± 0.03 ^d^*	8.57 ± 0.01 ^d^*
	1 d	83.70 ± 0.03 ^b^*	−3.23 ± 0.02 ^c^*	13.13 ± 0.06 ^c^*
	3 d	80.88 ± 0.01 ^c^*	−2.94 ± 0.05 ^b^*	17.64 ± 0.02 ^b^*
	7 d	76.37 ± 0.01 ^d^*	−2.64 ± 0.04 ^a^*	20.73 ± 0.03 ^a^*
AX-0.2%	0 d	86.60 ± 0.09 ^a^*	−4.23 ± 0.02 ^d^*	6.68 ± 0.03 ^d^*
	1 d	84.66 ± 0.02 ^b^*	−4.07 ± 0.02 ^c^*	10.41 ± 0.02 ^c^*
	3 d	81.25 ± 0.05 ^c^*	−3.91 ± 0.04 ^b^*	14.25 ± 0.03 ^b^*
	7 d	78.37 ± 0.01 ^d^*	−3.76 ± 0.05 ^a^*	17.84 ± 0.02 ^a^*

Note: Data are expressed as mean ± standard deviation; different lowercase letters indicate significant differences among different storage times within the same group (*p* < 0.05); * indicates significant difference compared with the blank control group at the same storage time (*p* < 0.05); L*: Lightness; a*: Red-green index; b*: Yellow-blue index.

**Table 5:** Sensory quality scores of MP with or without AX during refrigerated storage at 4 °C for 0, 1, 3, and 7 d.

	Storage Time	Color (25 Points)	Odor (25 Points)	Texture (25 Points)	Mouthfeel (25 Points)	Total Score (100 Points)
Blank control	0 d	17.6 ± 2.3 ^a^	21.6 ± 1.8 ^a^	19.8 ± 2.5 ^a^	18.8 ± 1.3 ^a^	77.8 ± 5.7 ^a^
	1 d	17.1 ± 1.5 ^a^	19.4 ± 2.6 ^ab^	17.6 ± 2.6 ^ab^	17.2 ± 2.3 ^ab^	71.3 ± 6.6 ^ab^
	3 d	16.1 ± 2.5 ^a^	17.0 ± 2.4 ^bc^	16.1 ± 2.9 ^bc^	16.1 ± 2.3 ^bc^	65.3 ± 8.5 ^bc^
	7 d	15.0 ± 3.0 ^a^	15.2 ± 3.0 ^c^	14.4 ± 2.6 ^c^	13.9 ± 3.2 ^c^	58.5 ± 10.0 ^c^
AX-0.1%	0 d	18.9 ± 2.0 ^a^	20.6 ± 2.3 ^a^	21.7 ± 2.5 ^a^	20.6 ± 2.3 ^a^	81.8 ± 5.8 ^a^
	1 d	19.0 ± 2.1 ^a^	20.6 ± 2.1 ^a^	21.0 ± 2.4 ^a^*	19.8 ± 1.7 ^a^*	80.4 ± 5.8 ^a^*
	3 d	16.9 ± 2.0 ^a^	17.7 ± 1.7 ^b^	17.4 ± 2.5 ^b^	18.1 ± 2.2 ^ab^	70.1 ± 6.3 ^b^
	7 d	16.8 ± 2.2 ^a^	16.5 ± 2.3 ^b^	15.3 ± 2.3 ^b^	15.9 ± 3.4 ^b^	64.5 ± 8.7 ^b^
AX-0.2%	0 d	18.6 ± 2.1 ^ab^	20.0 ± 3.2 ^a^	19.4 ± 4.7 ^ab^	19.1 ± 4.1 ^ab^	77.1 ± 12.5 ^a^
	1 d	19.2 ± 2.0 ^a^*	20.3 ± 1.8 ^a^	21.6 ± 2.1 ^a^*	21.2 ± 1.9 ^a^*	82.3 ± 5.4 ^a^*
	3 d	17.7 ± 1.8 ^ab^	18.7 ± 1.3 ^ab^	18.2 ± 2.1 ^a^	19.5 ± 1.8 ^ab^*	74.1 ± 5.2 ^ab^*
	7 d	16.3 ± 2.4 ^b^	16.6 ± 2.3 ^b^	17.1 ± 2.4 ^b^*	17.7 ± 3.1 ^b^*	67.7 ± 8.2 ^b^

Note: Data are expressed as mean ± standard deviation; different lowercase letters indicate significant differences among different storage times within the same group (*p* < 0.05); * indicates significant difference compared with the blank control group at the same storage time (*p* < 0.05).

## Data Availability

The original contributions presented in this study are included in the article/Supplementary Materials. Further inquiries can be directed to the corresponding authors.

## Supplementary Materials

The following supporting information can be downloaded at: https://www.mdpi.com/article/10.3390/foods15122212/s1, Table S1: Sensory evaluation criteria for MP; Table S2: Texture properties of freshly prepared laboratory-prepared MP (0 d) and commercial products; Table S3: Color parameters of freshly prepared laboratory-prepared MP (0 d) and commercial products; Table S4: Sensory quality of freshly prepared laboratory-prepared MP (0 d) and commercial products.
