# Supplementary material for: Arabinoxylan Improves Quality and Inhibits Starch Retrogradation in Mashed Potatoes Under Cold Storage
Source: Foods. 2026 Jun 19;15(12):2212. doi: 10.3390/foods15122212 (PMC13297698; doi:10.3390/foods15122212)
Supplement: Supplementary file 1 [file foods-15-02212-s001.zip › foods-4331953-supplementary.pdf]

Supplementary contents

Table S1. Sensory evaluation criteria for MP.

Item	Score	Scoring Criteria
Color (25 points)	21-25	Golden yellow, uniform color and lustrous
	16-20	Pale yellow, relatively uniform color and weak luster
	≤15	White or brown, uneven color and non-lustrous
Odor (25 points)	21-25	Natural mellow aroma of potato, no off-odor
	16-20	Weak potato aroma, no off-odor
	≤15	No potato aroma, with off-odor
Texture (25 points)	21-25	Uniform and delicate paste, easy to stir, no agglomeration, no impurities
	16-20	Relatively uniform and delicate paste, easy to stir, slight agglomeration, no impurities
	≤15	Non-uniform and coarse paste, difficult to stir, obvious agglomeration, with impurities
Mouthfeel (25 points)	21-25	Uniform and delicate mouthfeel, non-sticky, easy to chew, long aftertaste
	16-20	Relatively uniform and delicate mouthfeel, slightly sticky, easy to chew, weak aftertaste
	≤15	Rough and granular mouthfeel, sticky, difficult to chew, no aftertaste

Table S2. Texture properties of freshly prepared laboratory-prepared MP (0 d) and commercial products.

	Hardness (g)	Springiness	Cohesiveness	Gumminess	Chewiness	Resilience
Blank control	143.54 ± 13.34^{d}	0.97 ± 0.00^{b}	0.93 ± 0.04^{a}	133.78 ± 17.54^{d}	130.22 ± 16.83^{d}	0.05 ± 0.00^{bc}
AX-0.1%	119.45 ± 11.33^{d}	0.98 ± 0.00^{b}	0.83 ± 0.02^{a}	99.02 ± 8.02^{de}	96.51 ± 7.77^{de}	0.04 ± 0.00^{d}
AX-0.2%	73.43 ± 2.28^{d}	0.98 ± 0.00^{b}	0.83 ± 0.02^{a}	60.67 ± 1.61^{e}	59.19 ± 1.71^{e}	0.04 ± 0.00^{cd}
Commercial Product 1	724.66 ± 22.06^{b}	0.98 ± 0.01^{b}	0.54 ± 0.08^{b}	389.77 ± 66.80^{b}	379.79 ± 63.25^{b}	0.05 ± 0.00^{b}
Commercial Product 2	1138.46 ± 99.61^{a}	0.98 ± 0.00^{ab}	0.51 ± 0.07^{b}	576.41 ± 30.30^{a}	563.67 ± 29.11^{a}	0.07 ± 0.01^{a}
Commercial Product 3	478.61 ± 34.53^{c}	0.98 ± 0.00^{a}	0.57 ± 0.10^{b}	272.16 ± 31.80^{c}	267.75 ± 31.68^{c}	0.05 ± 0.01^{b}

Note: Data are expressed as mean ± standard deviation; different lowercase letters indicate significant differences among different samples ($p < 0.05$).

Table S3. Color parameters of freshly prepared laboratory-prepared MP (0 d) and commercial products.

	L^*	a^*	b^*
Blank control	90.43 ± 0.25^{a}	-2.49 ± 0.05^{b}	11.50 ± 0.17^{d}
AX-0.1%	88.35 ± 0.14^{c}	-3.41 ± 0.23^{c}	9.70 ± 0.02^{e}
AX-0.2%	86.88 ± 0.86^{d}	-4.53 ± 0.14^{e}	7.48 ± 0.24^{f}
Commercial Product 1	88.39 ± 0.58^{bc}	-1.92 ± 0.07^{a}	39.71 ± 0.17^{b}
Commercial Product 2	89.26 ± 0.10^{b}	-5.09 ± 0.16^{f}	43.95 ± 0.05^{a}
Commercial Product 3	77.27 ± 0.11^{e}	-3.87 ± 0.21^{d}	28.10 ± 0.47^{c}

Note: Data are expressed as mean ± standard deviation; different lowercase letters indicate significant differences among different samples ($p < 0.05$).

Table S4. Sensory quality of freshly prepared laboratory-prepared MP (0 d) and commercial products.

	Color (25 points)	Odor (25 points)	Texture (25 points)	Mouthfeel (25 points)	Total Score (100 points)
Blank control	18.1 ± 2.0^{c}	20.4 ± 1.7^{a}	21.1 ± 2.0^{a}	20.9 ± 1.1^{a}	80.5 ± 5.5^{a}
AX-0.1%	19.7 ± 1.3^{bc}	19.9 ± 1.9^{a}	21.5 ± 2.1^{a}	21.4 ± 1.6^{a}	82.5 ± 5.4^{a}
AX-0.2%	19.6 ± 1.6^{bc}	20.0 ± 1.7^{a}	21.5 ± 2.3^{a}	20.3 ± 1.5^{a}	81.4 ± 5.1^{a}
Commercial Product 1	21.5 ± 1.4^{a}	19.4 ± 3.1^{a}	20.2 ± 2.5^{a}	19.5 ± 1.5^{a}	80.6 ± 6.4^{a}
Commercial Product 2	21.3 ± 1.3^{ab}	19.1 ± 2.9^{a}	19.9 ± 2.1^{a}	19.8 ± 1.7^{a}	80.1 ± 6.3^{a}
Commercial Product 3	22.2 ± 1.4^{a}	20.6 ± 0.7^{a}	19.4 ± 2.5^{a}	21.2 ± 2.4^{a}	83.4 ± 5.1^{a}

Note: Data are expressed as mean ± standard deviation; different lowercase letters indicate significant differences among different samples ($p < 0.05$).
